# Comparison of Hemodynamic Response between Patients with Systolic Heart Failure Differing in Serum Aldosterone Concentrations during and after a 6-Minute Walk Test

**DOI:** 10.3390/jcm12031007

**Published:** 2023-01-28

**Authors:** Kamila Miętkiewska-Szwacka, Tomasz Krauze, Katarzyna Barecka, Anna Różańska-Kirschke, Dagmara Przymuszała-Staszak, Agata Schneider, Miłosz Dziarmaga, Jacek Lech Tarchalski, Aneta Nowak, Mateusz Bryl, Jolanta Kaczmarek, Jarosław Piskorski, Andrzej Wykrętowicz, Przemysław Guzik

**Affiliations:** 1 Department of Internal Medicine, Metabolic Disorders, and Hypertension, Poznan University of Medical Sciences, 60-786 Poznan, Poland; 2 Department of Cardiology—Intensive Therapy, Poznan University of Medical Sciences, 60-355 Poznan, Poland; 3 Department of Physiotherapy, Stanisław Staszic State University of Applied Sciences in Piła, 64-920 Pila, Poland; 4 Central Analytical and Biochemical Laboratory, Heliodor Święcicki Clinical Hospital, 60-355 Poznan, Poland; 5 Institute of Physics, University of Zielona Gora, Campus A, 65-516 Zielona Gora, Poland

**Keywords:** aldosterone, arterial blood pressure, exercise, heart failure, post-exercise recovery, 6 min walk test

## Abstract

Aldosterone regulates hemodynamics, including blood pressure (BP), and is involved in the development and progression of cardiovascular diseases, including systolic heart failure (HF). While exercise intolerance is typical for HF, neither BP nor heart rate (HR) have specific characteristics in HF patients. This study compares BP and HR profiles during and after standardized exercise between patients with systolic HF with either lower or higher aldosterone concentrations. We measured BP and HR in 306 ambulatory adults with systolic HF (left ventricular ejection fraction (LVEF) <50%) during and after a 6 min walk test (6MWT). All patients underwent a resting transthoracic echocardiography, and venous blood samples were collected for biochemical analyses. The patients were also divided into tertiles of serum aldosterone concentration: T1 (<106 pg/mL), T2 (106 and 263 pg/mL) and T3 (>263 pg/mL), respectively. Individuals from T1 and T2 were combined into T1–T2 as the reference group for comparisons with patients from T3. The individuals from T3 had significantly lower systolic, mean and diastolic BPs at rest, at the end and at 1 and 3 min post-6MWT recovery, as well as a more dilated left atrium and right ventricle alongside a higher concentration of N-terminal pro-B-type natriuretic peptide (NT-proBNP). Higher serum aldosterone concentration in HF patients with an LVEF < 50% is associated with a lower 6MWT BP but not an HR profile.

## 1. Introduction

The renin-angiotensin-aldosterone system (RAAS) is a hormonal cascade that regulates arterial blood pressure (BP), fluid volume and the sodium–potassium balance [1,2,3]. RAAS activity increases during heart failure (HF) with reduced ejection fraction (HFrEF) [3]. Aldosterone, a mineralocorticoid, binds with its receptors in distal kidney tubules, cardiomyocytes and vascular smooth muscle cells. It regulates the cardiovascular system’s (CVS) structural and functional changes and impacts the remodeling and fibrosis of the heart and arteries.

Aldosterone’s effects on the function of the CVS include sodium reabsorption, water retention and regulation of hemodynamics such as systemic vascular resistance, cardiac output and BP [1]. In regards to heart rate (HR), the data is uncertain [4]; some studies show no direct effect of aldosterone on HR, whereas others report that an aldosterone antagonist blockade may reduce the morning HR rise or improve HR response to exercise [4,5,6].

Typically, HF patients have poor exercise tolerance, assessed, for instance, by the distance covered on a flat, hard surface during a 6 min walk test (6MWT) [2,7]. BP and HR are measured during and after the 6MWT to monitor the primary hemodynamic response to exercise.

Usually, aldosterone concentration and HR are increased in HF patients [8], while BP does not have a specific behavior in HF [9]. In practice, aldosterone, BP and HR can be low, normal or increased during this disease. If aldosterone regulates hemodynamics, then both BP and HR might be related to its concentration in HF patients. Nevertheless, it is unknown whether such an association exists between aldosterone concentration and resting and exercise-induced hemodynamic changes in patients with HF. In this study, we aim to compare BP and HR profiles between patients with systolic HF with lower and higher aldosterone concentrations during a 6MWT.

## 2. Materials and Methods

### 2.1. Study Design

This research is a post-hoc analysis of a prospective, cross-sectional observational study enrolling ambulatory patients with stable systolic HF, diagnosed and treated according to the clinical guidelines of the European Society of Cardiology current to the enrolment time, i.e., 2010–2014 [2]. Patients were recruited for the project “Predicting adverse clinical outcomes in patients with implanted defibrillating devices” (grant TEAM/2009-4/4), which the Foundation for Polish Science funded within the TEAM program [10]. One author had full access to all the data in the study and took responsibility for its integrity and confidentiality.

The study was conducted in accordance with the Declaration of Helsinki [11]. The study protocol was reviewed and approved by The Bioethical Committee at Poznan University of Medical Sciences (approval no. 363/10 in 2009), Poznan, Poland. Informed written consent was obtained from all patients before enrolment.

Initially, we recruited 457 HF patients with implanted cardiac devices from the Outpatient Clinic of Cardiology Heliodor Swiecicki Clinical Hospital, University of Medical Sciences, Poznan. All patients had a previously implanted cardiac device—either an ICD (implantable cardioverter-defibrillator) or a CRT-D (cardiac resynchronization therapy defibrillator). For this substudy, we used the following additional inclusion criteria: (1) the presence of a significant left ventricular systolic dysfunction defined as reduced LVEF <50%; and (2) available results of both 6MWT and aldosterone serum concentration. Finally, 306 patients were selected for further analysis.

### 2.2. Clinical Assessment

All participants underwent a detailed clinical evaluation that included (1) current symptoms and past medical history, which provided information about days following the implantation of their ICD or CRT-D, their current pharmacological treatment, NYHA class, and other comorbidities; (2) measurement of body mass and height and derived body mass index (BMI); (3) performance of resting 12-lead electrocardiography (ECG) for identifying the type of rhythm, i.e., sinus, paced or atrial fibrillation/flutter, or other, determining the mean HR.

### 2.3. 6-Minute Walk Test

The 6MWT was performed according to the American Thoracic Society as the last examination during their visit [12]. Patients walked along a straight 32-metre flat corridor, with the final distance measurement being noted and calculated within a 1-metre accuracy when the patient finished the test. The 6 min walk test was performed as the last examination during the patient’s visit. The following parameters were measured: systolic BP (SBP), diastolic BP (DBP), mean BP (MBP), pulse pressure (PP) and HR at rest at the end of the 6MWT and 1 and 3 min after the exercise during recovery. All measurements were made seated on both arms using the oscillating method (Omron M3, Kyoto, Japan), and the higher values of both measures were employed for the analysis. The MBP was calculated using the 1/3 (SBP-DBP) + DBP formula. The PP was calculated as the difference between the systolic and diastolic BP: PP = SBP-DBP. Continuous oxygen saturation (SpO2) was monitored to detect the minimal SpO2 via a 2500A Pulse Oximeter (Nonin Medical INC, Plymouth, USA). The Borg scale estimated the maximal Rate of Perceived Exertion (RPE) to reflect the subjective experience of the physical activity intensity immediately after the walking part of the 6MWT was completed. The RPE ranges from 6 to 20, where 6 means “no exertion at all” and 20 means “maximal exertion” [12].

### 2.4. Biochemical Analysis

Venous blood samples were collected after overnight fasting, after obtaining all agreements, and before any other tests were performed. All blood samples were instantly transferred to the Central Laboratory of the Heliodor Swiecicki University Hospital in Poznan, Poland. The following biochemical tests were carried out on the same day: fasting blood creatinine, sodium, potassium and N-terminal pro-B-type natriuretic peptide. Aldosterone concentration was measured and stored at −80 °C degrees for the serum samples (DRG^®®^ Aldosterone ELISA, DRG International Inc., Springfield, IL, USA). The estimated glomerular filtration rate (eGFR) was calculated using the Cockcroft–Gault equation.

### 2.5. Transthoracic Echocardiography

Patients underwent resting transthoracic echocardiography (either Acuson CV70; Siemens, Munich, Germany or MyLab 30 CV; Esaote, Genova, Italy) using ultrasound transducers ranging from 1–4 MHz. According to the guidelines current to the enrolment time (by the American Society of Echocardiography and the European Association of Echocardiography), standard parameters were measured, such as ventricular thickness, chamber sizes, systolic and diastolic function, including LVEF via the biplane Simpson method, as well as E/A and E/e’, respectively [13].

### 2.6. Dividing Patients into Low and High Serum Aldosterone Concentration Groups

The serum aldosterone concentration in healthy individuals can vary depending on many factors, and the threshold separating abnormal values can differ between studies and authors. In patients with cardiac conditions such as hypertension or HF, the impact of therapy and comorbidities such as diabetes on the RAAS can also play a significant role [1,2,3,5,6]. As a result, clear thresholds for abnormal serum aldosterone concentrations have not been established in patients with systolic HF.

**Figure 1:**
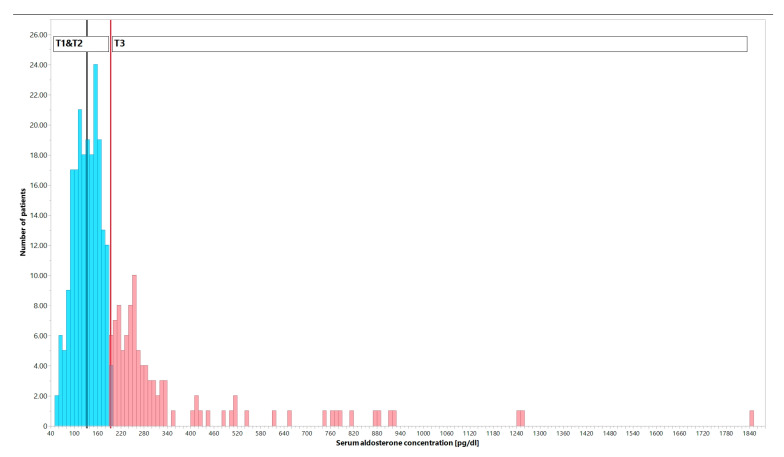
Distribution of the number of patients with systolic heart failure (HF) according to various serum aldosterone concentrations. Patients were divided into tertiles of serum aldosterone concentrations: first tertile (T1) refers to patients within the range of 56–133 pg/dL, second tertile (T2) to the range of 134–193 pg/dL, and third tertile (T3) to the range of 194–1840 pg/dL. The T1 and T2 groups were combined into a lower serum aldosterone concentration group (*n* = 204, represented by blue bars) for comparison with the T3 group (*n* = 102, represented by red bars).

To address this lack of a threshold, we divided patients with systolic HF into three groups (tertiles) (Figure 1): T1, T2 and T3. The serum aldosterone concentration was between 56 and 133 pg/dL in T1, 134 and 193 pg/dL in T2, and 194 to 1840 pg/dL in T3. Our primary objective was to compare patients with systolic HF who had lower vs higher serum aldosterone concentrations. To do this, we combined individuals from T1 and T2 into one group, T1–2, which represented the lower aldosterone group, and compared them to the T3 group, which represented the higher aldosterone group.

### 2.7. Statistical Analysis

Most continuous and discrete data did not have a normal distribution through the Shapiro–Wilk test. Therefore, these data were described as median and the 25th and 75th percentiles. The Mann–Whitney test was used to compare continuous data between patients from the combined T1–T2 and T3 groups. The Friedman test for repeated measures was utilized to study the effects of the 6MWT and recovery on BP profiles. Qualitative data is shown as numbers meeting the given criterion and a relative value as a percentage. The comparisons for the qualitative data were carried out with either the Fisher exact test or Pearson’s chi-squared test.

Our primary intention was to compare the HR and BP profile during the 6MWT and clinical features between patients with lower and higher aldosterone concentrations. The activity of RAAS, including aldosterone concentration, may be modified by various factors, including treatment and clinical covariates [1,2,3]. As our findings show (please refer to the Results Section), there were significant differences in the rate of spironolactone use and renal function between the T1–T2 and T3 groups, which might bias the interpretation. To overcome these issues, we applied case-control matching to obtain patients with an identical distribution of spironolactone use and comparable kidney function. We used two criteria for the matching: (1) an exact match for the rate of spironolactone and (2) the difference in creatinine concentration of no more than 0.5 mg/dL between patients from the T1–T2 and T3 groups. Next, the results were compared with the paired nonparametric Wilcoxon and the exact Fisher tests for continuous/discrete and qualitative data, respectively. These results are shown in the Supplementary Materials as not originally related to the study’s aim.

Statistical analyses were performed using the MedCalc Statistical Software version 19.1 (MedCalc Software by Ostend, Ostend, Belgium; 2019), PQStat v.1.8.4.152 (PQStat Software, Poznan, Poland), and JMP Pro 17.0.0 (SAS Institute Inc., Cary, NC, USA). The results were obtained using two-sided tests, and significance was set at *p* < 0.05.

## 3. Results

### 3.1. Comparison of Baseline Characteristics between T1–T2 and T3 Patients

The first tertile (T1) included patients with the lowest serum aldosterone concentration (106 (93.25–119) pg/mL), the second tertile (T2) comprised those with intermediate serum aldosterone concentration (158.5 (148–171.75) pg/mL) and the third tertile (T3) contained patients with the highest serum aldosterone concentration, i.e., above 263 (229.25–336.5) pg/mL. A summary and comparisons of the baseline clinical characteristics of the patients from the T1–T2 (*n* = 204) and T3 (*n* = 102) groups are shown in Table 1 for continuous and discrete data and Table 2 for the qualitative data. 

Indeed, comparable in the T1–T2 and T3 individuals were the following: age, BMI, NYHA class, pre-test resting SpO2, RPE during the 6MWT, potassium blood concentrations and most echocardiographic measures, including LVEF and the thickness of the left ventricle (LV) walls. However, patients from the T3 aldosterone group had significantly higher creatinine and NT-proBNP, lower sodium concentrations, and worse eGFR. Additionally, they had a larger left atrium (LA) and right ventricle (RV) and a shorter distance covered during the 6MWT. Regarding the qualitative data, people from the T3 group had a lower rate of ischemic origin of HF but more commonly had CRT-D rather than only ICD, aldosterone receptor antagonists and diuretics.

### 3.2. Comparison of Hemodynamic Profiles between T1–T2 and T3 Patients

Most participants were men. SpO2 did not decline <94% in any studied patient during the 6MWT. The patients in the T3 group achieved significantly higher (two-fold) serum aldosterone concentration whilst also exhibiting lower serum sodium values than the T1–2 group. Moreover, the T3 group reported significantly increased NT-proBNP concentrations, creatinine levels and lower eGFR results. The T3 patients were characterized by a significant dilation of the left atrium and right ventricle. There was no difference in the LVEF and E/e’ results. However, the T3 group presented a trend towards larger LVDD. Patients from the T3 group had significantly less-common ischaemia-related HF. 

The incidence of hypertension (HA), diabetes mellitus type 2 (DM2), smoking (ex- or current smoker), previous stroke/ transient ischemic attack (TIA) or type of atrial fibrillation (AF) was comparable between the T1–2 and T3 groups. However, the T3 patients had significantly more frequently implanted CRT-D (nearly 13%) and need for aldosterone receptor antagonists (over 16%) or a diuretic (over 12%) than did the remaining individuals. No other differences in the therapies were found. 

In both groups, BP and HR displayed a physiological response during the test, elevating during exercise and returning to baseline values during the post-exercise recovery (Table 3). However, the T3 group patients had lower pre-6MWT values of SBP, DBP, MBP and PP. Regardless of the BP level, the profiles of dynamic changes in SBP, DBP, MBP and PP during exercise are comparable in patients with lower (T1–T2) and higher (T3) aldosterone concentrations. 

## 4. Discussion

We found that patients with HF and an LVEF <50% as well as a higher (T3) aldosterone concentration had, compared to other individuals (T1–T2), lower SBP, MBP and DBP, but no significant difference in HR before and at the end of the 6MWT or during the post-6MWT recovery. Their PP was significantly lower only during the post-exercise recovery. These patients also had a higher plasma NT-proBNP concentration, more dilated LA and RA, covered a shorter distance during the 6MWT, and exhibited worse kidney function. They were treated more frequently with diuretics, including spironolactone, and more commonly had an implanted CRT-D. Regardless of the aldosterone concentration, the profile of the BP response to exercise during the 6MWT and post-exercise recovery was comparable in all HF patients.

### 4.1. Clinical Implications and Prognostic Value of Low Blood Pressure in Patients with Stable HFrEF

SBP and HR typically increase in healthy adults during exercise, whereas DBP decreases or remains unchanged [14]. Diniz et al. [15] studied survivors of an acute myocardial infarction with a median LVEF of 54% and observed similar physiological HR and BP increases during and 5 min after the 6MWT. Although our patients had a lower LVEF of approximately 31%, their HR and BP profiles were similar to healthy people and post-infarction patients. Nevertheless, the HF subjects with a higher aldosterone concentration had constantly lower BP and PP but similar HR when compared to individuals with lower aldosterone concentration. 

In the OPTIMISE-HF study, patients hospitalized with an acute HF and SBP < 120 mm Hg at admission frequently had LV systolic dysfunction and a worse prognosis [16]. Lee et al. presented an association between SBP <100 mmHg and mortality amongst patients with an LVEF ≤ 45% and NYHA II and III functional classes [17]. They also reported that a SBP of ~110 mmHg separates patients into those who would or would not benefit from the current HF treatment. Ather et al. [18] found that a lower resting BP was more frequently associated with a worse prognosis in patients with systolic HF. 

Most drugs routinely used in HF treatment decrease BP, e.g., ACE inhibitors, angiotensin II receptor blockers, beta-blockers, nitrates or hydralazine, and diuretics, including aldosterone antagonists. Even modern pharmaceutical agents recommended for treating advanced systolic HF, such as sacubitril/valsartan and sodium-glucose co-transporter-2 inhibitors, reduce BP [19]. 

In general, HF patients with lower BP are worse at tolerating multidrug HF pharmaceutical treatments. Lower BP may lead to hypoperfusion of the coronary, cerebral, renal and muscle vasculature if SBP declines <110 mmHg. Patients with even lower SBP, i.e., <90 mmHg, have more severe organ hypoperfusion, and the co-existence of advanced or end-stage HF with low cardiac output worsens their clinical condition [20].

### 4.2. Neurohormonal Adaptation in Patients with Stable HFrEF

Several hemodynamic impairments accompanying LV systolic dysfunction in HF patients can cause compensatory activation of the sympathetic nervous system and RAAS, aiming at preserving cardiac output [2,3,4,5,21]. Lower organ and tissue blood perfusion causes hypoxia, stimulating chemoreflex and metaboreflex, which enhances the adrenergic drive and RAAS activation [22]. The impaired perfusion also activates various mechanoreflexes, increasing these responses even more [23,24]. 

Increased aldosterone release into circulation enhances sodium and water absorption and further progresses fluid congestion [1]. LV diastolic dysfunction, accompanying severe systolic HF, impairs venous return from the pulmonary circulation to LA, causing fluid congestion and the activation of various low-pressure receptors in the atria and veins [25]. An increased preload stretches and stimulates the cardiac chambers and their volumoreceptors, causing a rise in the release of natriuretic peptides. 

Our findings also show that patients with systolic HF and higher aldosterone have reduced or non-increasing BP. Reduced arterial BP unloads aortic and carotid baroreceptors and further stimulates adrenergic activity [26]. The long-term outcomes of these complex and prolonged sympathetic and RAAS activations are cardiomyocyte apoptosis and maladaptive ventricular and vascular remodeling. These pathophysiological processes further contribute to the progression of cardiac dysfunction [21,27]. 

HF patients also have an impaired metaboreflex from both working and respiratory muscles [24,28]. Due to worsening blood perfusion to the skeletal muscles, myopathy develops. Consequently, people with HF experience poorer exercise tolerance with quicker fatigue and dyspnea [29]. Ponikowski et al. [30] studied chronic HF patients with preserved exercise capacity. They found that the abnormal enhancement of the ventilatory response to exercise might indicate some disruption of the cardiorespiratory reflex control by hypoxic and hypercapnic chemosensitivity, autonomic cardiac control, baroreflex sensitivity and ergoreflex response. Exercise training improves functional capacity in HF patients and can potentially stop the progression of this vicious cycle in which RAAS is involved [24,31]. 

### 4.3. Pharmacological Impact

Over 80% of our patients were on a mineralocorticoid antagonist, exclusively spironolactone. At the time of the patients’ enrolment in our study, eplerenone was not reimbursed in Poland and thus practically unavailable for most patients. Most of our patients were also on ACE inhibitors or angiotensin II receptor blockers, and many were using diuretics. Spironolactone, ACE inhibitors/angiotensin II receptor blockers and diuretics strongly affect the RAAS and might influence the results of aldosterone concentration measurements [1]. Renal insufficiency also activates RAAS [1] and, as Oda et al. [32] suggested, eGFR < 60 mL/min/1.73 m^2^ further augments sympathetic activation in HF patients. 

Our study was cross-sectional and not designed to explore pathophysiology. Most of the mentioned mechanisms co-stimulate RAAS and increase aldosterone concentration in individuals with more severe systolic HF and lower BP. However, we cannot reckon which is more probable—whether higher aldosterone concentrations are responsible for the clinical findings or more advanced HF triggers various reflex mechanisms to increase serum aldosterone concentration. These considerations are further complicated by the effects of the applied multidrug pharmacological treatment modifying RAAS [1]. 

### 4.4. Limitations

Our findings refer to patients with advanced systolic HF with a median LVEF < 31%. Therefore, we cannot extrapolate these results to HF patients with better LV systolic functions. We conducted this study between 2010 and 2014; at that time, our HF patients were on optimal pharmacological and nonpharmacological (ICD or CRT-D) medications. 

Such drugs as angiotensin receptor-neprilysin inhibitors and sodium-glucose co-transporter 2 inhibitors are currently recommended in systolic HF pharmacotherapy. However, they were not available during the study period of the present project. For this reason, the extrapolation of our conclusions to patients treated with newer therapies might be limited.

Undoubtedly, drugs influencing RAAS and renal function may affect our findings. Patients with higher serum aldosterone were more frequently taking spironolactone (76.5% vs. 60.3%; *p* = 0.005), and had worse renal function (median creatinine concentration 1.16 vs 1.03 mg/dL; *p* = 0.0001). This cross-sectional study was conducted in consecutive ambulatory patients with systolic HF, an LVEF < 50% and an implanted ICD or CRT-D. We did not use any tighter inclusion or exclusion criteria, as the differences in the various medications and the presence of different comorbidities might also express some genuine clinical phenomena. Preserving the consecutive nature of enrolled patients in observational studies is important and seems to reflect real associations better. However, the apparent differences in the rate of spironolactone use and worse kidney function may bias our findings.

After applying case-control matching adjusted for the same rate of spironolactone use and comparable renal function (creatinine concentration differing between patients no more than 0.5 mg/dL), we repeated our analyses (Supplementary Materials). Most clinical differences between the lower and higher serum aldosterone groups disappeared. However, patients with higher aldosterone had significantly lower DBP and MBP at rest and during the 6MWT. This additional analysis supports our original findings that lower BP accompanies a higher aldosterone concentration in patients with advanced systolic HF. It is plausible then that higher aldosterone concentration is a reflex hormonal response of the RAAS to reduced BP and worse tissue and organ (including kidney) perfusion [1].

### 4.5. The Novelty of the Study and Potential Clinical Impact

Our study yielded two novel findings. First, HF patients with an LVEF < 50% and a higher aldosterone concentration appeared to have a more advanced disease accompanied by lower BP. Second, regardless of the lower BP profile, the dynamic changes in SBP, DBP, MBP and PP were comparable between patients with lower and higher aldosterone concentrations during exercise. 

Although speculative, if an HF patient with an LVEF < 50% presents with a lower BP, such a person probably has a higher serum aldosterone concentration. This information has potential future implications. 

First, aldosterone measurement might help identify patients with more advanced systolic HF. Second, as HF patients with higher aldosterone seem to have a more severe disease, more aggressive treatment with newer drugs influencing this mineralocorticoid directly or indirectly should be considered. Most of the available pharmacological agents modifying the RAAS reduce BP and are not well tolerated by hypotensive patients. Newer drugs affecting RAAS should probably be neutral for BP. 

Third, aldosterone concentration might help monitor the clinical progress and effectiveness of the applied therapy in patients with systolic HF with an LVEF < 50%. We are aware that these are only speculations that deserve future investigation.

## 5. Conclusions

Higher serum aldosterone concentration is accompanied by lower BP at rest, during exercise and post-exercise in patients with HF and an LVEF < 50%. Such individuals also have a more dilated left atrium and right ventricle, increased NT-proBNP concentration, worse kidney function, and cover a shorter distance during the 6MWT.

## Figures and Tables

**Table 1:** Comparison of baseline clinical characteristics of patients with HF and LVEF <50% and implanted defibrillating device divided into subgroups of higher (T3) and lower (T1–T2) serum aldosterone concentration. Data are presented as median and (25–75P), and comparisons were made with the Mann–Whitney test.

	T1 and 2 *n* = 204		T3 *n* = 102		*p*-Value
	Median	25–75 P	Median	25–75 P	
Age, years	63.24	57.94–68.58	65.73	57.15–73.96	0.1348
BMI, kg/m^2^	28.31	25.43–31.0	27.94	24.51–31.37	0.6651
NYHA class	2.0	2.0–3.0	2.0	2.0–3.0	0.9373
Weeks from ICD/CRT-D implantation	17	5.0–30.0	19	9.0–34.00	0.2853
Aldosterone, pg/mL	133.5	106.00–158.50	263	229.00–337.00	<0.0001
Crea, mg/dL	1.03	0.89–1.27	1.16	1.00–1.49	0.0001
eGFR_C-G_, mL/min/1.73 m^2^	82.561	61.64–107.88	69.667	49.89–92.70	0.0016
NT-proBNP, pg/mL	811.9	374.40–2378.50	1145	485.30–2783.00	0.0425
K, mmol/L	4.52	4.27–4.78	4.495	4.30–4.87	0.9676
Na, mmol/L	141	139.00–143.00	140	138.00–142.00	0.0006
LVEDD, mm	60.6	55.0–66.95	63.2	57.30–71.20	0.0581
LVESD, mm	50.9	44.60–58.85	54.25	45.90–61.50	0.0583
LA, mm	43.15	39.30–47.60	45.85	39.70–50.30	0.0315
RV, mm	27.8	25.40–31.60	29.15	26.20–32.90	0.0385
IVT, mm	11.1	9.75–12.35	11.2	9.60–12.70	0.911
LVPWT, mm	11.6	10.20–13.20	11.25	9.60–13.20	0.3759
E/A ratio	0.78	0.60–1.55	1.018	0.60–2.23	0.1308
E/e’ ratio	8.13	5.85–11.20	8.53	5.62–11.84	0.7131
E-wave velocity, cm/s	62.5	49.50–92.00	67	47.75–96.25	0.4113
LVEF, %	30.93	23.39–37.44	30.93	21.33–36.72	0.4371
Distance, m	480	384.00–544.00	432	320.00–512.00	0.0303
RPE	12	10.00–15.00	12	10.00–15.00	0.7746
Resting SpO2, %	96	95.00–97.00	96	95.00–98.00	0.2715

Abbreviations: BMI, body mass index; Crea, creatinine; CRT-D, cardiac resynchronization therapy defibrillator; E-wave velocity, peak early diastolic LV inflow; E/e’, the ratio of E to the velocity (e’) of myocardial tissue at the base of the mitral annulus during early diastole; eGFR_C-G,_ estimated glomerular filtration rate by Cockcroft–Gault equation; ICD, implantable cardioverter-defibrillator; IVT, intraventricular septum thickness; K, potassium; LA, left atrium; LVEDD, left ventricle end-diastolic diameter in the parasternal long-axis view; LVEF, left ventricular ejection fraction by the biplane Simpson method; LVESD, left ventricle end-systolic diameter in the parasternal long-axis view; LVPWT, left ventricle posterior (basal segment of the inferolateral) wall thickness, Na, sodium; NT-proBNP, N-terminal pro-B-type natriuretic peptide; NYHA, New York Heart Association I-IV; resting SpO2, resting stable oxygen saturation. RPE, Borg Rating of Perceived Exertion Scale; RV, right ventricle.

**Table 2:** Baseline medical and medicine characteristics of participants in both groups.

	T1 and 2		T3		*p*-Value
	*n*	%	*n*	%	
Men	177	86.8	86	84.3	0.5615
Ischaemic aetiology	103	50.5	37	36.3	0.0188
Hypertension	167	81.9	83	81.4	0.9169
Diabetes mellitus type 2	71	34.8	35	34.3	0.9324
Current smoker status	32	15.7	13	12.7	0.0659
Stroke/TIA	20	9.8	10	9.8	1.0000
Permanent AF	42	20.6	24	23.5	0.6086
NYHA III/IV	70	34.3	36	35.3	0.8653
Primary/Secondary	38	18.6	13	12.7	0.1938
CRT-D	80	39.2	53	52.0	0.0343
ACE or ARBs	170	83.3	83	81.4	0.6697
Beta-blocker	176	86.3	85	83.3	0.4942
Spironolactone	123	60.3	78	76.5	0.0050
Diuretic	164	80.4	94	92.2	0.0077
Statin	163	79.9	81	79.4	0.9200
Digoxin	15	7.4	10	9.8	0.4613
Nitrate	23	11.3	7	6.9	0.2219
Antiplatelet drug	155	76.0	78	76.5	0.9246
Amiodarone	43	21.2	23	22.5	0.7685
Insulin	17	8.3	7	6.9	0.6525

Abbreviations: ACE, Angiotensin-converting enzyme inhibitor; AF, atrial fibrillation; ARBs, Angiotensin II receptor blocker; CRT-D, cardiac resynchronization therapy defibrillator; NYHA, New York Heart Association III/IV; TIA, transient ischemic attack.

**Table 3:** Comparisons between patients from T1–T2 and T3 groups at rest, the end of the 6 min walk test (6MWT), and 1 and 3 min post-exercise recovery for systolic blood pressure (SBP), diastolic blood pressure (DBP), mean blood pressure (MBP), pulse pressure (PP) and heart rate (HR) in both groups. Data are presented as median and (25–75P), and comparisons were made with the Mann–Whitney test.

	T1 and 2		T3		*p*-Value
	Median	(25–75P)	Median	(25–75P)	
SBP_rest_, mmHg	136.00	121.00–148.00	127.00	115.00–143.00	0.005
SBP_end_, mmHg	144.00	126.00–160.00	139.00	121.00–152.00	0.0147
SBP_recov-1′_, mmHg	141.00	125.00–156.00	130.00	117.00–146.25	0.0008
SBP_recov-3′_, mmHg	137.00	124.50–154.00	133.00	112.75–143.25	0.0013
DBP_rest_, mmHg	84.50	77.00–92.00	81.00	71.00–89.00	0.0082
DBP_end_, mmHg	88.00	80.00–95.50	84.00	74.00–93.00	0.0235
DBP_recov-1′_, mmHg	87.00	80.00–95.00	81.00	74.00–94.00	0.0012
DBP_recov-3′_, mmHg	86.00	79.00–94.00	83.00	73.00–90.00	0.0017
MBP_rest_, mmHg	101.50	93.17–111.83	97.00	87.33–105.67	0.0036
MBP_end_, mmHg	106.50	97.33–117.00	102.00	91.67–111.67	0.0099
MBP_recov-1′_, mmHg	105.00	95.50–114.50	97.17	89.00–109.00	0.0001
MBP_recov-3′_, mmHg	104.33	95.17–113.67	96.50	87.00–106.33	0.0001
PP_rest_, mmHg	50.00	40.00–60.00	46.00	37.00–57.00	0.1324
PP_end_, mmHg	55.00	40.00–66.50	52.00	39.00–61.00	0.1687
PP_recov-1′_, mmHg	51.50	41.00–63.00	47.00	36.00–60.00	0.0465
PP_recov-3′_, mmHg	50.00	39.00–61.50	47.50	35.00–58.00	0.086
HR_rest_, mmHg	71.00	64.00–78.00	71.00	65.00–81.00	0.6297
HR_end_, mmHg	89.00	76.50–101.50	85.00	77.00–100.00	0.5114
HR_recov-1′_, mmHg	76.00	69.00–87.50	76.00	68.00–88.50	0.9571
HR_recov-3′_, mmHg	72.50	66.50–84.00	75.00	68.00–86.00	0.4278

Abbreviations: DBP, diastolic blood pressure; HR, heart rate; MBP, mean blood pressure; PP, pulse pressure; SBP, systolic blood pressure; 6MWT, 6-min walk test; ‘0’, start of the 6MWT; end, end of the 6MWT; ‘1’ and ‘3’ post-exercise recovery.

## Data Availability

The datasets generated and/or analyzed for this study are currently not publicly available due to further ongoing analyses by the authors. Selected data, however, are available from the corresponding author upon request.

## Supplementary Materials

The following supporting information can be downloaded at: https://www.mdpi.com/article/10.3390/jcm12031007/s1. **Table S1**. Comparison of continuous and descrete data of clinical characteristics of patients with HF and LVEF <50% and implanted defibrillating device divided into subgroups with lower (<195.0 pg/mL) and higher (>195.0 pg/mL)serum aldosterone concentration. **Table S2**. Comparison of qualitative and nominal data of patients with HF and LVEF <50% and implanted defibrillating device divided into subgroups with lower (<195.0 pg/mL) and higher (>195.0 pg/mL) serum aldosterone concentration.

## Abbreviations

6MWT	6 min walk test
ACE	angiotensin-converting enzyme inhibitor
AF	atrial fibrillation
ARBs	angiotensin II receptor blocker
BMI	body mass index
BP	blood pressure
CVS	cardiovascular system
Crea	creatinine
CRT-D	cardiac resynchronization therapy defibrillator
DBP	diastolic blood pressure
E-wave velocity	peak early diastolic LV inflow
ECG	electrocardiography
eGFR_C-G_	estimated glomerular filtration rate by Cockcroft–Gault equation
HA	hypertension
HF	heart failure
HFrEF	heart failure with reduced ejection fraction
HR	heart rate
ICD	implantable cardioverter-defibrillator
IVT	intra-ventricular septum thickness
K	potassium
LA	left atrium
LV	left ventricle
LVEDD	left ventricle end-diastolic diameter in the parasternal long-axis view
LVEF	left ventricular ejection fraction by the biplane Simpson method
LVESD	left ventricle end-systolic diameter in the parasternal long-axis view
LVPWT	left ventricle posterior (basal segment of the inferolateral) wall thickness
MBP	mean blood pressure
Na	sodium
NT-proBNP	N-terminal pro-B-type natriuretic peptide
NYHA	New York Heart Association I-IV
PP	pulse pressure
RAAS	renin-angiotensin-aldosterone system
resting SpO2	resting stable oxygen saturation
RPE	Borg Rating of Perceived Exertion Scale
RV	right ventricle
SBP	systolic blood pressure
T1	first tertile
T2	second tertile
T3	third tertile
TIA	transient ischemic attack
