# Supplementary material for: Comparison of Hemodynamic Response between Patients with Systolic Heart Failure Differing in Serum Aldosterone Concentrations during and after a 6-Minute Walk Test
Source: J Clin Med. 2023 Jan 28;12(3):1007. doi: 10.3390/jcm12031007 (PMC9917580; doi:10.3390/jcm12031007)
Supplement: Supplementary file 1 [file jcm-12-01007-s001.zip › jcm-2144814-supplementary.pdf]

Supplementary material

Several factors influence serum aldosterone concentration, including mineralocorticoid antagonists and renal function. In crude, uncontrolled analysis, these parameters differ significantly between patients with systolic HF and lower (the combined lower and middle tertiles - T1&T2) and higher (third tertile - T3) serum aldosterone concentration.

To mitigate these effects, we performed case-control matching between patients from T1&T2 and T3 groups for the use of spironolactone (set at no difference) and creatinine concentration (up to a 0.5 mg/dl acceptable difference). From the 306 patients studied, we obtained two groups of matched patients, each with 85 individuals. The comparisons of continuous and discrete data by the paired nonparametric Wilcoxon test can be found in Table S1. The qualitative data were compared by the exact Fisher test and are shown in Table S2.

Table S1. Comparison of continuous and descrete data of clinical characteristics of patients with HF and LVEF <50% and implanted defibrillating device divided into subgroups with lower (<195.0 pg/ml) and higher (>195.0 pg/ml) serum aldosterone concentration. Patients from both groups are case-control matched using the criteria the same rate of spironolactone use and no more than 0.5 mg/dl difference in the creatinine concentration. Data are presented as median and (25th-75th percentiles), p-values come from the nonparametric paired Wilcoxon test.

	Serum aldosterone concentration				p-value
	< 194. pg/ml N=85		> 194.0 pg/ml N=85		
	Median	25 - 75 P	Median	25 - 75 P	
Age, years	63.42	56.92 - 70.04	64.60	56.17 - 73.33	0.9354
BMI, kg/m^2	29.15	25.75 - 31.97	28.18	25.17 - 31.75	0.7617
NYHA class	2.00	2.00 - 3.00	2.00	2.00 - 3.00	0.6308
weeks from ICD/CRT-D implantation	17.00	7.00 - 30.00	19.00	9.50 - 35.50	0.1497
Aldosterone, pg/ml	140.00	114.00 - 165.00	255.00	225.00 - 353.00	<0.001
Crea, mg/dl	1.08	0.98 - 1.31	1.08	0.99 - 1.31	0.7119
$eGFR_{C-G}$, ml/min/1,73 m^2	76.79	58.43 - 102.94	72.76	56.69 - 97.71	0.4645
NT-proBNP, pg/ml	751.40	3340.75 - 2182.25	900.70	436.33 - 2076.25	0.6597
K, mmol/l	4.53	4.20 - 4.78	4.46	4.30 - 4.80	0.4894
Na, mmol/l	141.00	139.00 - 143.00	140.00	138.00 - 142.00	0.0026
LVEDD, mm	60.70	54.75 - 67.55	63.00	57.25 - 71.05	0.1117
LVESD, mm	51.50	43.58 - 59.05	53.30	45.52 - 61.03	0.2350
LA, mm	43.10	39.41- 48.70	44.30	39.30 - 50.13	0.3384
RV, mm	27.40	25.50 - 31.90	28.80	26.18 - 32.53	0.2109
IVT, mm	11.70	9.80 - 12.90	11.20	9.65 - 12.90	0.2940
LVPWT, mm	12.00	10.58 - 13.43	11.60	9.88 - 13.40	0.2591
E/A ratio	0.75	0.60 - 1.66	0.94	0.56 - 1.79	0.3179
E/e' ratio	7.78	5.56 - 10.61	8.00	5.19 - 11.13	0.5064
LVEF, %	30.03	22.95 - 36.81	31.11	22.60 - 36.94	0.8729
E-wave velocity, cm/s	60.00	48.00 - 90.25	65.00	47.00 - 92.50	0.5012
Distance, m	448.00	352.00 - 544.00	448.00	380.50 - 512.00	0.6648
RPE	12.00	10.00 - 15.00	12.00	10.00 - 15.00	0.9365
Resting SpO2, %	96.00	95.00 - 97.00	96.00	96.00 - 98.00	0.0760
SBP_{rest}, mmHg	136.00	119.75 - 148.25	127.00	116.00 - 143.75	0.0671
SBP_{end}, mmHg	141.00	123.00 - 160.00	139.00	125.00 - 152.25	0.3146
$SBP_{recov-1'}$, mmHg	141.00	122.75 - 158.00	131.00	117.75 - 147.00	0.0608
$SBP_{recov-3'}$, mmHg	135.00	124.75 - 155.25	134.00	115.00 - 145.00	0.1118
DBP_{rest}, mmHg	86.00	77.00 - 95.25	81.00	71.75 - 89.00	0.0031

DBP_{end}, mmHg	89.00	80.75 - 97.00	83.00	74.00 - 93.00	0.0175
$DBP_{recov-1'}$, mmHg	87.00	78.75 - 98.25	81.00	74.00 - 94.00	0.0292
$DBP_{recov-3'}$, mmHg	87.00	79.00 - 97.00	82.00	73.75 - 91.00	0.0252
MBP_{rest}, mmHg	103.00	92.00 - 113.58	96.67	87.25 - 105.17	0.0084
MBP_{end}, mmHg	107.33	94.83 - 118.00	101.33	92.17 - 111.83	0.0566
$MBP_{recov-1'}$, mmHg	105.67	93.33 - 116.17	97.33	89.92 - 108.75	0.0197
$MBP_{recov-3'}$, mmHg	104.33	95.25 - 115.00	99.33	88.75 - 106.75	0.0434
PP_{rest}, mmHg	47.00	36.75 - 58.25	47.00	41.00 - 57.50	0.7696
PP_{end}, mmHg	51.00	37.00 - 65.00	52.00	41.75 - 62.00	0.6734
$PP_{recov-1'}$, mmHg	51.00	38.00 - 64.00	48.00	37.50 - 60.50	0.5309
$PP_{recov-3'}$, mmHg	51.00	39.00 - 61.25	50.00	35.75 - 59.50	0.5580
HR_{rest}, mmHg	73.00	64.00 - 82.00	70.00	64.00 - 80.25	0.4046
HR_{end}, mmHg	87.00	75.75 - 103.50	85.00	77.00 - 100.00	0.9024
$HR_{recov-1'}$, mmHg	79.00	69.00 - 87.25	76.00	70.00 - 88.50	0.5758
$HR_{recov-3'}$, mmHg	76.00	68.00 - 83.00	75.00	68.00 - 86.00	1

Abbreviations: BMI, body mass index; Crea, creatinine; CRT-D, cardiac resynchronization therapy defibrillator; DBP, diastolic blood pressure; E-wave velocity, peak early diastolic LV inflow; E/e' - the ratio of E to the velocity (e') of myocardial tissue at the base of the mitral annulus during early diastole; $eGFR_{C-G}$ estimated glomerular filtration rate by Cockcroft-Gault equation; HR, heart rate; ICD, implantable cardioverter-defibrillator; IVT, intraventricular septum thickness; K, potassium; LA, left atrium; LVEDD, left ventricle end-diastolic diameter in the parasternal long-axis view; LVEF, left ventricular ejection fraction by the biplane Simpson method; LVESD, left ventricle end-systolic diameter in the parasternal long-axis view; LVPWT, left ventricle posterior (basal segment of the inferolateral) wall thickness, MBP, mean blood pressure; Na, sodium; NT-proBNP, N-terminal pro-B-type natriuretic peptide; NYHA, New York Heart Association I-IV; PP, pulse pressure; SBP, systolic blood pressure; resting SpO2, resting stable oxygen saturation; RPE, Borg Rating of Perceived Exertion Scale; RV, right ventricle; 6MWT, 6-minute walk test; '0', start of the 6MWT; end, end of the 6MWT; '1' and '3' of the post-exercise recovery.

Table S2. Comparison of qualitative and nominal data of patients with HF and LVEF <50% and implanted defibrillating device divided into subgroups with lower (<195.0 pg/ml) and higher (>195.0 pg/ml) serum aldosterone concentration. Patients from both groups are case-control matched using the criteria of the same rate of spironolactone use and no more than 0.5 mg/dl difference in the creatinine concentration. Data are presented as numbers and %, p-values come from the the exact Fisher test.

	Serum aldosterone concentration				p-value
	< 158.5 pg/ml N=153		> 158.5 pg/ml N=153		
	N	%	N	%	
Men	70	82.35	77	90.59	*0.1165*
Ischaemic aetiology	31	36.47	42	49.41	*0.0883*
Hypertension	68	80.00	73	85.88	*0.3080*
Diabetes mellitus type 2	25	29.41	32	37.65	*0.2554*
Current smoker status	11	12.94	11	12.94	*1.0000*
Stroke/TIA	9	10.59	11	12.94	*0.6340*
Permanent AF	17	20.00	18	21.18	*0.8496*

NYHA III/IV	49	32.03	57	37.25	*0.3373*
Primary/Secondary	9	10.59	12	14.12	*0.4844*
CRT-D	44	51.76	37	43.53	*0.2824*
ACE or ARBs	72	84.71	73	85.88	*0.8286*
Beta-blocker	68	80.00	75	88.24	*0.1419*
Spironolactone	67	73.82	67	73.82	*1*
Diuretic	78	91.76	82	96.47	*0.1923*
Statin	63	74.12	71	83.53	*0.1332*
Digoxin	9	10.59	7	8.24	*0.5994*
Nitrate	6	7.06	13	15.29	*0.0884*
Antiplatelet drug	66	77.65	69	81.18	*0.5704*
Amiodarone	18	21.18	20	23.53	*0.7127*
Insulin	3	3.53	8	9.41	*0.1190*

Abbreviations: ACE, Angiotensin-converting enzyme inhibitor; AF, atrial fibrillation; ARBs, Angiotensin II receptor blocker; CRT-D, cardiac resynchronization therapy defibrillator; NYHA, New York Heart Association III/IV; TIA, transient ischemic attack;

Following case-control matching of patients with lower and higher serum aldosterone, the rate of spironolactone use and kidney function were comparable. No significant differences were found for most continuous and discrete data between patients with lower and higher aldosterone concentrations. However, patients in the higher serum aldosterone group had significantly lower DBP at all phases of the 6MWT and MBP at the pre-test stage and 1 and 3 minutes post-test completion.

In conclusion, higher aldosterone levels are associated with reduced DBP and MBP in patients with systolic HF, regardless of spironolactone use and kidney function.
